# Virtual Reality for Aggression Assessment: The Development and Preliminary Results of Two Virtual Reality Tasks to Assess Reactive and Proactive Aggression in Males

**DOI:** 10.3390/brainsci11121653

**Published:** 2021-12-17

**Authors:** Jill Lobbestael, Maaike J. Cima

**Affiliations:** 1 Department of Clinical Psychological Science, Faculty of Psychology and Neuroscience, Maastricht University, 6211 Maastricht, The Netherlands; 2 Department Developmental Psychopathology, Brain Science Institute, Radboud University, 6525 Nijmegen, The Netherlands; maaike.cima@ru.nl

**Keywords:** aggression, assessment, reactive aggression, proactive aggression, virtual reality

## Abstract

Validly measuring aggression is challenging because self-reports are plagued with biased answer tendencies and behavioral measures with ethical concerns and low ecological validity. The current study, therefore, introduces a novel virtual reality (VR) aggression assessment tool, differentially assessing reactive and proactive aggression. Two VR tasks were developed, one in an alley environment (*N* = 24, all male, M_age_ = 23.88, 83.3% students) and an improved second one in a bar (*N* = 50, all male, M_age_ = 22.54, 90% students). In this bar VR task, participants were randomly assigned to either the reactive condition where they were triggered by a cheating and insulting dart-player or to the proactive condition where they could earn extra money by aggressing. Participants’ level of self-reported aggression and psychopathy was assessed, after which they engaged in either the reactive or proactive VR task. Changes in affect and blood pressure were also measured. Aggression in the reactive VR task was evidenced to mostly display convergent validity because it positively correlated with self-reported aggression and total and fearless dominance factor scores of psychopathy, and there was a trend relationship with increased systolic blood pressure. The validity of the proactive aggression variant of our VR bar paradigm received less support, and needs more refinement. It can be concluded that VR is a potentially promising tool to experimentally induce and assess (reactive) aggression, which has the potential to provide aggression researchers and clinicians with a realistic and modifiable aggression assessment environment.

## 1. Introduction

Aggression refers to behavior directed toward another with the intention to cause harm that the other wants to avoid [1]. Although a differentiation between types of aggression can be based on the form of expression (e.g., verbal or physical), a motivational-based distinction is particularly informative because it refers to the origins of aggression and, therefore, designates avenues for therapeutic interventions. One of the most common motivational differences of aggression is that of reactive versus proactive. Reactive aggression refers to ‘hot-blooded’ uncontrolled or impulsive outbursts of anger that serve as a defensive reaction to provocation or frustration. In contrast, proactive aggression is relatively non-emotional and ‘cold-blooded’, often premeditated or planned, typically used to gain extrinsic benefits such as money or power [2]. Individuals can engage in both types of aggression, which should be considered as separate dimensions [3]. Research has linked both aggression types to distinct developmental precursors and genetic factors, as well as behavioral and psychopathological concepts [4,5,6,7].

### 1.1. Current Available Aggression Measures and Their Drawbacks

Empirical studies mostly operationalize aggression in adults through self-report, like the Reactive-Proactive Questionnaire [8] and the Impulsive/Premeditated Aggression Scale [9]. Main drawbacks of self-report, however, are that aggression is highly socially undesirable and people often lack insight into their aggression levels [10,11]. In order to bypass these disadvantages, behavioral aggression paradigms were developed. The most often used behavioral aggression tasks include the Competitive Reaction Time Task (CRTT) [12] where the duration and intensity of aversive noise-blasts provided via a headphone that participants can administer to an opponent who is slower on a reaction time task reflects aggression, the Point Subtraction Aggression Paradigm [13] where participants can steal points from another player in a computer game, who will lose money because of this, and the Hot Sauce Paradigm [14] which indexes aggression via the amount of extremely spicy sauce provided to another participant who dislikes hot food. One crucial and common feature of aggression paradigms is that they provide participants with the opportunity to negatively impact an alleged opponent. An additional advantage of behavioral aggression tasks is that they provide the opportunity to actually expose participants to aggression-triggering situations, instead of merely inquiring for aggressive tendencies in a hypothetical way, as is the case in self-report.

There is quite some empirical evidence for adequate psychometric properties of several behavioral aggression paradigms [14,15,16,17,18]. Likewise, behavioral aggression tasks showed meaningful associations with established aggression correlates, such as violence in offenders [19], reactivity after provocation [15], alcohol [16,20], and viewing violent scenes [21].

Nonetheless, existing behavioral paradigms are plagued by four main disadvantages. First, behavioral aggression paradigms mostly present their participants with methods that are quite abstract or unrelated to violence in daily life (e.g., providing someone with hot sauce). Therefore, they are often criticized for their lack of ecological validity [22]. Second, they typically require elaborate cover stories. Participants are, e.g., told that they will compete against an opponent in an adjunct lab, but extra effort is needed to render the cover story sufficiently believable, like including a second experimenter and/or confederate participants or incorporating technical messages in the tasks. Oftentimes, the believability of the cover story will depend on details and on the acting work of the experimenter. A literature overview [23] shows that, even despite extensive effort, around 10% of participants drop out because of disbelief in the cover story, yielding unnecessary efforts and costs. Additionally, the use of cover stories is not optimal from an ethical standpoint and requires careful debriefing. A third disadvantage is that most aggression paradigms only assess general aggression, without differentiating between reactive and proactive motivations. Although there are some exceptions to this (e.g., the CRTT and the Tilt/Noise task [12,24]), where the proactive aggression conditions can indeed be distinguished from their reactive counterparts by the lack of provocation, it is arguable though whether winning a game in both these tasks is an ecologically valid alternative to the motivation for proactive aggression in real life, i.e., gaining money, resources, power, or prestige [25]. A final drawback of existing behavioral aggression paradigms is that they fail to assess the most readily available form of aggression, i.e., a direct physical attack of another individual. Because of obvious ethical reasons, available paradigms instead rely on proxies of human victims (i.e., inflatable dolls in the Bobo Modeling Paradigm [26], voodoo dolls in the Voodoo Doll Task [27], or body opponent bags [28]) or on proxies of aggressive acts (i.e., administering aversive white noise or hot sauce). One exception here is the Bungled Procedure Paradigm [29] where participants are given the opportunity to shoot a human target with a pellet or paintball gun. In reality, participants never actually get to shoot at the target though; hence, this task merely measures potential aggressive intentions [30]. Although it is obvious that these restrictions are needed because of ethical reasons, the concern here is again one of ecological validity.

### 1.2. Virtual Reality as a Potential Tool to Measure Aggression

One particularly promising candidate tool to validly assess behavioral aggression is virtual reality (VR). Applied to psychology, VR is an advanced form of three-dimensional human–computer interaction in which individuals can freely move and interact [31]. The most basic VR technology consists of desktop computers and gaming devices which create a two- or three-dimensional virtual environment [32]. The more recent and common VR setups, however, consist of a head-mounted display (HMD): a helmet with an integrated liquid crystal display screen for each eye, generating a wide and stereoscopic view of the virtual environment [33]. A tracking system analyzes where the HMD is positioned and how it is oriented so that the computer can instantly update the virtual environment as if the person would move in the real world [34]. The tracking system can also use sensors attached to the participant’s hand in order to present these in the virtual world. Next to images, relevant three-dimensional sounds can be presented, either via earphones or via speakers in the room, aiding in more realistic feelings for the user. Consequently, a sense of presence or immersion develops, implying that participants (at least partly) forget that they are in a laboratory [31].

While fairly newly applied in research on psychopathology, VR has been shown useful in behavioral assessment (e.g., of stress [35,36], schizophrenia [37,38], food cravings [39], and autism [40]).

VR has mostly been used to stimulate aggression in the context of 3D violent video games. Here, participants are instructed to kill as many opponents as possible through shooting them with a gun. Outcomes included increased aggressive or hostile feelings [41,42,43,44,45,46], physical arousal [44], or aggressive behavior operationalized as the number of killings [45,46] or aversive noise administration to opponents in the Competitive Reaction Time Task [41]. These 3D violent video games can be considered to assess aggression in general, instead of reactive or proactive aggression, as the shooting is unrelated to provocation and there are no aggression incentives other than winning the game. Having participants emerged in violent killing games considerably lacks ecological validity as an aggression outcome, since it is highly unlikely that people get shot at with a gun in real life. Furthermore, previous exposure to regular violent video games might have desensitized participants when in a similar 3D VR environment, especially amongst frequent players [47,48,49]. Lastly, killing others in such VR tasks could also assess competitiveness rather than aggression [50,51].

A handful of other VR studies used more realistic and common anger-triggering situations. Some presented participants with anger-triggering scenes, such as an office setting with a criticizing supervisor [52,53]. In a mixed gender general sample from the general population, one study found partial support that these scenes increased heart rate and self-reported anger, although not at all time points [52]. In the second study, including mixed gender (retired) soldiers, these scenes were found to significantly increase anger [53]. After students were exposed to unnerving sounds of heavy construction work, they reported a significantly stronger increase in anger compared to after being exposed to a joyful environment, although these environmental VR tasks did not lead to differential physiological reactivity [54]. Another study had their participants interact with one or more virtual agents who at some point became aggressive, with the instruction to deescalate the aggression, and the results showed that, although this VR scene impacted electrodermal activity less compared to observing a real person becoming aggressive, the physiological responses in this VR scene followed a similar path [55]. One final study had the participants observe VR scenes with one aggressor and one victim to assess the bystander effect, in a scene where two avatars had a conversation about football, which resulted in one verbally threatening the other and pushing him to the wall [22]. The latter study focused on feasibility though, and no specific outcomes were provided. While all these VR designs presented participants with suitable triggers for reactive aggression, only one [55] involved an interaction between real participants and avatars, none have been fully validated, and aggression did not constitute the outcome measure of these studies. Taken together, VR has not been previously used to differentially trigger and measure reactive and proactive aggression.

There are several potential advantages to using VR technology in the behavioral assessment of aggression. Firstly, VR scenarios can be constructed in such a way that cover stories are unnecessary, thereby lowering dropout due to disbelievers. Secondly, no actual harmdoing is involved, making it free of ethical constraints, and providing the opportunity to even assess physical aggression. Thirdly, in addition to being highly standardized (and, thus, experimentally controlled), VR environments are highly modifiable, allowing aggression assessment in different environments, in response to different avatars (e.g., race, gender) and of different motivations. Lastly, when compared to the typical laboratory environment, participants are likely to experience more privacy in VR due to immersion despite the presence of the experimenter. Similarly, since VR participants get the illusion that what is happening is real, their mental and physical responses are more likely to mirror those of an actual similar situation [22]. Both these factors increase the likelihood of participants presenting real-life behavior.

### 1.3. The Present Study 

The main goal of the current study was to develop a valid VR assessment tool to differently trigger and assess both reactive and proactive aggression. Validity was assessed by comparing the VR reactive and proactive aggression outcomes with their relevant self-reported aggression levels. Convergent validity was further determined by assessing their link to self-reported levels of psychopathy, a personality constellation marked by manipulation, arrogance, a lack of empathy, adult antisocial behavior, and impulsiveness that has been strongly linked to aggression [56,57]. Specifically, meta-analytic evidence showed psychopathy to be related to both reactive and proactive aggression [58]. The current study presents the development of two different aggression VR tasks in two consecutive studies, to illustrate the development process. Realistic environments were used in both VR paradigms (i.e., an alley and a bar), with a high likelihood of being related to real-life occurrence of aggression. To our best knowledge, this is the first VR study to directly measure aggression in the VR context in a physical form (i.e., by hitting the avatar). The advantage of this approach is that participants get the opportunity to aggress while emerged in the VR, as opposed to aggression being assessed in a subsequent and nonrelated behavioral task [41]. 

## 2. Study 1

### 2.1. Materials and Methods

#### 2.1.1. Sample 

A total of *N* = 24 male single students aged between 18 and 52 (M_age_ = 23.88, SD = 7.07) participated in this study. Power analysis shows that a minimum of *N* = 21 participants was needed the detect a large effect size of |*p*| = 0.50, power 80%. Only males with a basic comprehension of the Dutch language were included. People with epilepsy and severe motion sickness were excluded from participation. The majority had a Dutch nationality (75%), 20.8% were German, and 4.2% were of other nationalities. With respect to educational level, 58.3% finished high school, 12.5% finished intermediate vocational education, 20.8% finished higher vocational education, and 8.3% finished university. Most participants (83.3%) were attending university, while 16.7% were working. Only male participants were recruited because men generally exhibit higher levels of aggression [59] and psychopathy [60,61,62,63,64,65,66] than women. This allowed us to avoid confounding gender effects, as well as possible floor effects.

#### 2.1.2. Measures

**Figure 1:**
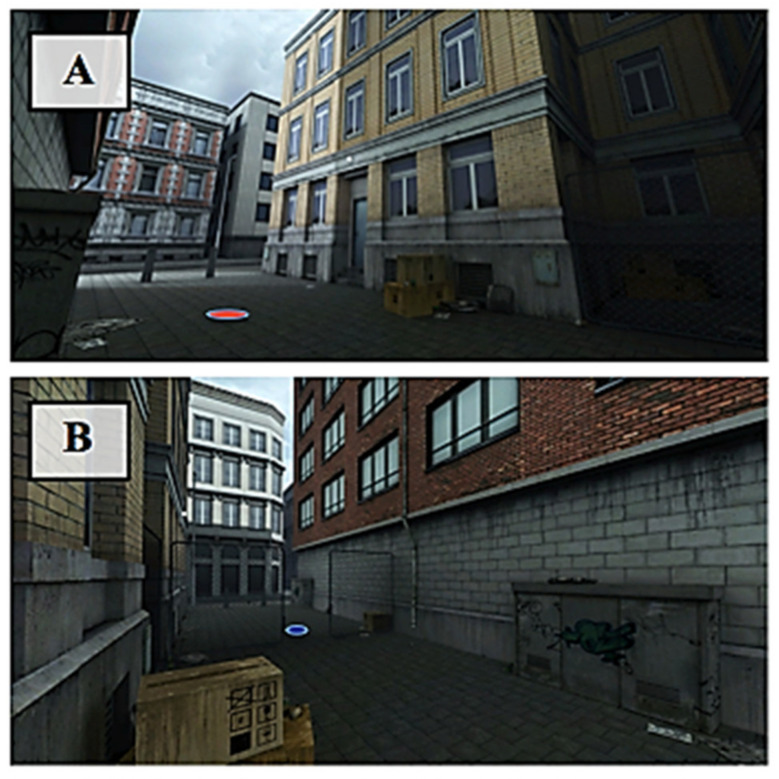
(**A**) Virtual environment of the participant’s side of the fence; the red spot indicates the participant’s approximate starting position; (**B**) virtual environment from the opposite angle; the blue spot indicates the approximate goal position in the proactive condition.

**Figure 2:**
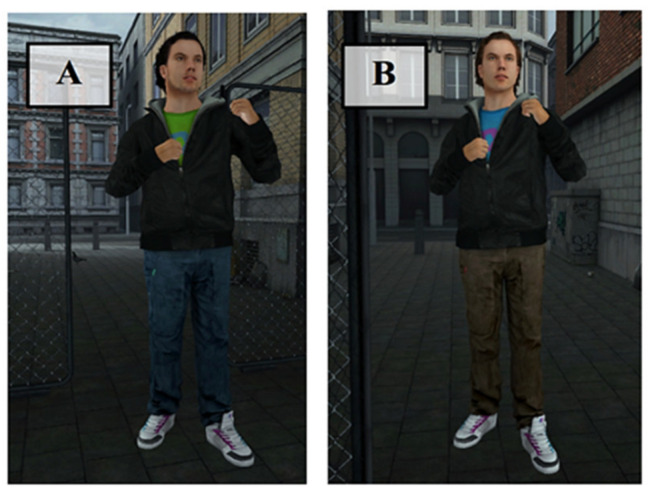
(**A**) Avatar in proactive condition; (**B**) avatar in reactive condition.

**Figure 3:**
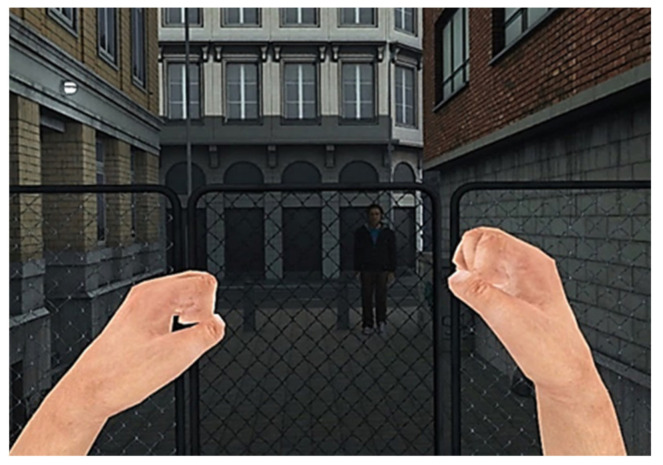
Virtual representation of the participants’ hands.

Two versions of the VR task were designed: one assessing reactive aggression, and one assessing proactive aggression. The VR scenery was that of a small city alley ranked by two house walls on the left and right and by houses in front and behind. A chain-link fence with a centered passageway was located in the middle of the alley, thereby dividing the alley into two parts. The virtual environment was further designed to realistically resemble a city side alley by the addition of a distribution box, cardboard boxes, and some litter (see Figure 1). Background noise was attuned to the scenario (e.g., sounds of cars driving by, children playing, background chatter). We opted for a male avatar because the threshold for male individuals to use violence against the same sex has been shown to be lower than against the opposite sex [67]. The avatar’s characteristics (length, hair color) were similar to those of the average male Dutch population (see Figure 2). The avatars of both conditions looked quite alike, but wore different clothes; one was bearded and the other was not to ensure that both avatars would be comparable in characteristics, yet distinguishable between conditions. Participants were able to see and use a virtual representation of their hands when they moved them in front of them. The fingers of these virtual hands were semi-closed and were not flexible (see Figure 3). After a brief exploration phase, participants were asked to step on the starting position, a red spot on the floor (see Figure 1A), after which an avatar appeared on the other side of the fence walking toward the participant. In the proactive condition, the virtual individual opened the door in the fence and positioned himself in the passageway, thereby blocking it. The participants’ task in this condition was to reach the other side of the fence (marked by a blue spot on the floor, see Figure 1B). Participants were instructed not to just go through the virtual individual, but to only pass the doorway once the avatar stepped aside. Participants did not receive any further instructions. In order to make the avatar step aside, participants were required to aggress against him by striking him either in the head or chest. The avatar briefly turned red to signal a successful hit. Twenty seconds after the first successful hit the avatar stepped aside. This, however, remained unknown to the participants, leaving open the possibility to both initiate and continue aggressing against the avatar within these 20 s. The number of strikes in this condition was used as a measure of proactive aggressive behavior. The idea was that aggressive behavior in this condition would be used to reach a goal (the blue spot), not as response to provocation. For the reactive condition, participants were not given any specific instructions. Instead, they were asked to react in a way that they deemed appropriate for the given situation. In this condition, the avatar did not stop in the passageway, but continued walking toward the red spot (i.e., the starting position of the participant). The avatar stopped once he reached the red spot, at which point he started hitting the participant. In case the participant left the red spot for more than 20 s, the virtual individual turned around and walked away. Again, the participants had the possibility to aggress against the avatar by using their fists to hit the avatar. It was hypothesized that aggressive behavior in this condition would be an indication of reactive aggression since it arose due to a reaction toward provocation (i.e., being hit by the avatar).

Self-reported aggression was assessed using the Reactive–Proactive Questionnaire (RPQ [8], Dutch translation [68]). The RPQ consists of 23 items, of which 11 measure reactive and 12 measure proactive aggression. Items are rated on a three-point Likert scale ranging from 0 = “never” to 2 = “often”. Prior studies reported good test–retest reliability and construct and criterion validity [8,68], and evidenced via factor analyses that the two-factor solution outperformed the one-factor solution [8,68,69]. In the present sample, α = 0.72 for reactive aggression, α = 0.64 for proactive aggression, and α = 0.79 for total aggression.

Psychopathy traits were assessed using the Dutch version of the Psychopathic Personality Inventory Revised (PPI-R [70,71]). Although there is some debate about the underlying factor structure of the psychopathy construct, there is good support for the triarchic model of psychopathy [72]. This model identifies boldness (including dominance, emotional stability, and venturesomeness), disinhibition (deficient inhibitory control), and meanness (callousness and aggressive resource seeking) as core psychopathic features. The triarchic model has also received support in psychopathy research using nonclinical samples, which typically relies on assessment instruments excluding reference to criminal and antisocial behavior [73]. One example is the Psychopathy Personality Inventory-revised (PPI-R [70]), which consists of the three factors of fearless dominance, self-centered impulsivity, and cold-heartedness. Psychopathy (in particular the boldness factor [74]) has been previously shown to relate to both increased reactive [75] and proactive aggression [5,76], while the disinhibition factor was uniquely related to reactive aggression [74]. The PPI-R is a self-report questionnaire consisting of 154 items that have to be scored using a four-point Likert scale (1 = false, 2 = mostly false, 3 = mostly true, and 4 = true). Factor analysis has indicated two main factors: fearless dominance (FD) and self-centered impulsivity (SCI). Seven out of eight subscales of the PPI load on these two scales, i.e., fearlessness, social potency, stress immunity, Machiavellian egocentricity, carefree nonplanfulness, blame externalization, and impulsive nonconformity, respectively. The eighth factor of cold-heartedness did not load on either of these two factors [77]. Satisfactory internal validity (PPI-R total, α = 0.91; PPI FD, α = 0.91; PPI SCI, α = 0.89, PPI cold-heartedness, α = 79), construct validity (correlates ranging from *r* = 0.18–0.68 with other psychopathy measures), and external validity were reported in prior studies for PPI-R factors [71], as well as high test–retest reliability (PPI-R total, *r* = 0.93; PPI-R FD, *r* = 0.91; and PPI-R SCI, *r* = 0.90 [78]). In the present sample, α = 0.86 for PPI-R total, α = 0.61 for PPI FD, α = 0.78 for PPI SCI, and α = 0.76 for PPI cold-heartedness.

Seven questions were asked to assess how immersed participants were in the VR environment (1. How nauseous/sick did you feel after the experiment? 2. How genuine did the VR world feel? 3. How genuine did the avatar look? 4. To what degree did you have the feeling of being immersed in the VR world? 5. If 0 means “*I was in the lab the whole time*” and 100 means “*I was in in the world*”, how did you feel during the VR task?). Participants were also asked to rate how threatening and friendly they perceived the avatar. All items had to be rated on a 100 mm visual analogue scale.

#### 2.1.3. Procedure

All measures, manipulations, and exclusions in the study are reported. This within-subject study was conducted in accordance with the Declaration of Helsinki, and the protocol was approved by the Ethical Committee of Maastricht University (approval code ECP 08_03_2013). Participants were recruited via advertisements at university billboards. Upon arrival at the VR lab, participants were informed about the general study procedures and signed informed consent. Next, participants were randomly assigned to either first conducting the reactive condition, followed by the proactive condition, or the other way around. The VR lab of Maastricht University is a room of 6 × 4 m equipped with 23 canton speakers and cameras that are part of the highly accurate Phasespace tracking system. The speakers were placed around the lab for a 360° sound experience, and the sound stimulation was run on a Mac desktop. Participants could move freely wearing a backpack with wireless receivers and a head-mounted display (HMD, Nvis ST-50) that provides a 3D stereoscopic view. The virtual environment automatically adjusts to the participant’s head motions and orientation. To run the virtual simulation, a Linux server was used to communicate between the various components, and an Intel Xeon windows computer with an Nvidia Quadro video card was used to render the simulation. The task was programmed in Python (via Vizard; WorldViz VR, Santa Barbara, CA, USA), while graphical content was made with Blender 3D and 3Ds Max (Motionbuilder; Autodesk, Mill Valley, CA, USA). Using this experimental setup, the participant was free to walk around, could locate sounds, and was able to explore the 3D environment. This helped to optimize immersion in the VR scenes. The VR scene was created by a digital artist. Before the start of the VR experiment, participants were aided with connecting the Nvis ST-50 VR helmet and a lightweight backpack. The backpack included the batteries and HDMI transmitters, since the experiment was conducted with a wireless connection. In order to visualize the participant’s hands, a Phasespace system was used which consisted of two motion sensing controllers, allowing both hands to be used individually. Instructions regarding the use of the devices were given to the participant. Participants then filled out the trait measures (i.e., PPI-R and RPQ), followed by the VR questionnaire. Finally, an exit interview was administered to verify the participants` ideas on the purpose of the study. Directly after the end of the experiment, the written debriefing was handed out and read aloud by the experimenter. On request of the ethical committee, it was explicitly noted that aggressive behavior is not tolerable or desirable in everyday situations. The participant was thanked for participating. Psychology undergraduates received credit points in return for their participation. All other participants received a monetary reward voucher.

### 2.2. Results

Table 1 provides the descriptive statistics of all study variables. The level of reported nausea was low (also when compared to other studies [79,80]). Authenticity of the VR environment and avatars and telepresence were rated between 17 and 47/100 and, thus, comparable to the results of some other studies [81,82], but lower than observed in other studies [79,80,83,84,85]. The avatars were perceived as slightly friendly and somewhat more threatening.

Six participants (25%) did not aggress in the reactive VR condition. The remaining 18 participants (75%) hit the provoking avatar between one and 274 times, M = 57.44, SD = 83.13, always after being attacked by the avatar. Because this count variable was strongly negatively skewed, we used negative binomial regression analyses with the number of hits rescaled to decimal categories as the dependent variable, and with self-reported aggression and psychopathy (subscales) as covariates. Table 2 shows that the degree of aggression displayed in the reactive aggression VR condition correlated significantly positively with total and reactive self-reported aggression, as well as with psychopathy total and cold-heartedness. In the proactive VR aggression condition, all participants (100%) aggressed. The mean number of hits ranged between two and 29 times, M = 11, SD = 9.32. Overall, the number of hits in the reactive condition was higher than that in the proactive condition, *t*(23) = 2.09, *p* = 0.048. Table 2 shows that the degree of aggression displayed in the proactive aggression condition did not significantly correlate with self-reported aggression or with psychopathy.

### 2.3. Discussion Study 1

One positive feature of this first alley version of a VR assessment tool for reactive and proactive aggression was that both conditions clearly differed in provocation level (unprovoked by the avatar in the proactive condition and provoked by the avatar who started to hit the participants in the reactive condition). Furthermore, the external validity of the reactive VR task was evidenced by its significant correlation with self-reported reactive aggression and psychopathy. 

However, several drawbacks of this alley VR task became apparent. In general, participants rated the authenticity of and immersion in this alley VR environment as rather low. Another drawback was the within-subject design, which primed the participants with aggression in the first condition, likely consequently increasing aggression in the second condition. Therefore, the second condition cannot be considered a pure, naturalistic assessment of aggression. One drawback of the reactive aggression condition was that it can be considered rather unrealistic that avatars simply started hitting someone out of the blue, as reported by several participants.

The proactive aggression condition turned out to have multiple drawbacks. Firstly, proactive VR aggression did not correlate with any of our self-report measures. Secondly, participants were not given any incentive to aggress other than reaching a blue spot. This might have lacked ecological validity as, per definition, proactive aggression is installed to obtain, e.g., money or prestige [2,25]. Thirdly, the avatar in the proactive condition only stepped aside in case participants hit him. As a related issue, there was nothing else to do in this proactive VR condition aside hitting. We also noticed that participants often spoke to the experimenter, asking questions or commenting on their own behavior, indicating lower VR emergence in this proactive condition. In addition, the experimenters observed that almost all participants considered other options before hitting the avatar, like trying to articulate their intentions by talking to the avatar, trying to gain his attention by waving at him, or trying to lure him away from the doorway. Therefore, the finding that all participants hit the avatar in the proactive condition was at least partly due to demand characteristics or avoiding boredom, as, in fact, proactive aggression is rather uncommon. Lastly, the avatar obstructed the way for the participant, which can to some extent be considered provocative, thereby making the proactive condition less ‘pure’. 

These limitations illustrate the need for a new VR aggression assessment task with improved visuals and VR emergence qualities, with a between-subject design to avoid learning effects. Additionally, participants needed to be provided with more background information as to why the avatar started to provoke them in the reactive aggression condition, to make it more realistic. The ecological validity of the proactive VR task needed to be improved by providing participants with alternative options asides from aggressing. All these issues were addressed in a second VR assessment tool, as outlined in study 2.

## 3. Study 2

The primary goal of this study was to develop a new VR assessment tool for reactive and proactive aggression based on what we learned from study 1 and to assess its feasibility. Importantly, a between-subject design was now used to avoid confounding effects of two consecutive behavioral aggression tasks. As was the case in study 1, study 2 assessed the relationship between the reactive and proactive aggression VR conditions with self-reported reactive and proactive aggression, as well as with (the subfactors of) psychopathy, to determine construct validity. 

The current study had three additional research questions, i.e., assessing the relationship between the reactive and proactive VR outcomes and: (1) the differential expression forms of aggression, i.e., verbal versus physical aggression, (2) changes in affect from baseline to post VR conditions, and (3) changes in blood pressure from baseline to post VR conditions. The expression forms of aggression were assessed under the hypothesis of a positive association between our VR task and physical aggression. Research questions (2) and (3) are based on the theoretical assumption that reactive and proactive aggression have differential effects on both affect and physiological arousal, i.e., reactive aggression is related to anger and physiological arousal, while this is not the case for ‘cold-blooded’, proactive aggression [2,87]. Several studies indeed evidenced reactive aggression to be related to increased anger [88,89,90], heart rate, skin conductance [86,88,89,91,92], and blood pressure [93]. In contrast, hyporesponsive heart rate, skin conductance [92,94,95], and blood pressure [93] following stress have been shown to relate to proactive aggression. However, there is also considerable variability across results as several studies did not find the expected differential relationships for reactive and/or proactive aggression regarding such biological markers [85,86,87,88,92,94,96].

### 3.1. Materials and Methods

#### 3.1.1. Sample

A total of *N* = 50 male students (*N* = 26 in the reactive condition, and *N* = 24 in the proactive condition) participated in this study. Power analysis showed that a minimum of *N* = 21 participants was needed to detect a large effect size of |*p*| = 0.50, power 80%. Only males with a basic comprehension of the Dutch language were included. People with epilepsy and severe motion sickness were excluded from participation. Table 3 shows an overview of the demographic variables of the total group and split by condition. Participants were aged between 18 and 30. The majority were Dutch undergraduate students, and about half were single. There were no significant differences in age, nationality, education, marital status, or working situation between participants of the reactive and proactive VR condition. Four percent of participants were on antidepressants (one in each condition), while the remainder of the participants were unmedicated. 

#### 3.1.2. Measures

**Figure 4:**
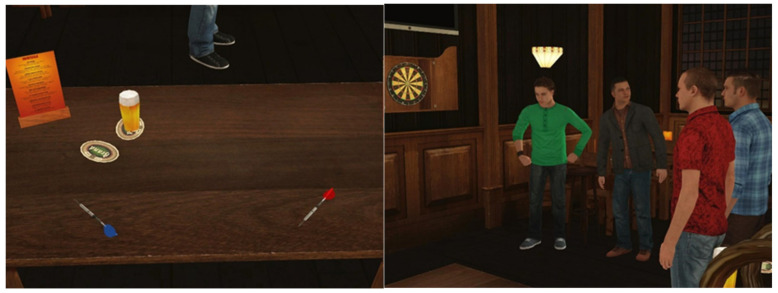
The VR pub scenery. **Left**: The table where the participant was sitting behind. **Right**: Dartboard, two players (read and blue shirts), and two spectators.

**Figure 5:**
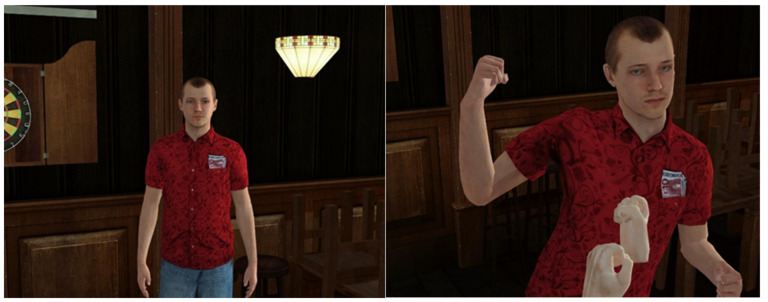
**Left**: The cheating avatar walking toward the participant in the reactive condition. **Right**: In case the participant hit the avatar, the avatar fought back. In front, a virtual display of the participant’s hands is displayed.

**Figure 6:**
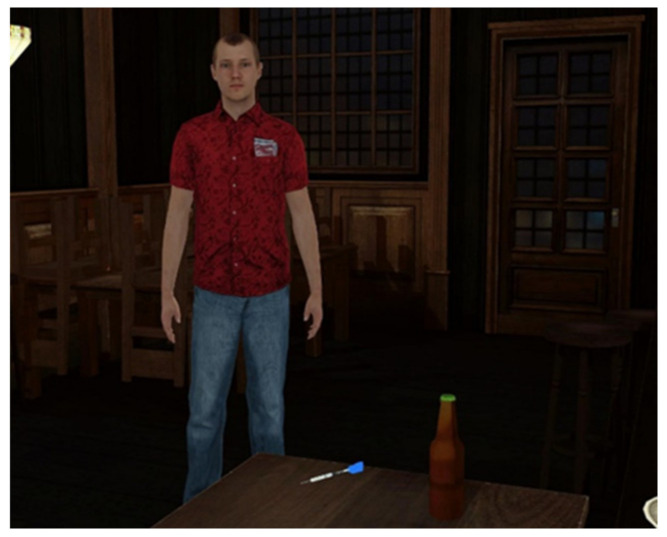
The second part of the proactive condition. The participant was presented with the options to either pick up the dart arrow and play a game, pick up the bottle and start hitting the avatar, or engage in a fistfight with the avatar.

Two versions of the VR task were designed: one assessing reactive aggression, and one assessing proactive aggression. The VR scenery was that of a pub. Background noise consisted of people talking, similar to that of a real pub. In part 1, participants were situated sitting behind a table (see Figure 4), on which there were two darts (one blue and one red). On the right side of the participant, a dartboard hanging on the wall and four male avatars standing in line next to the dartboard were visible. Two of these avatars (one with a blue and one with a red shirt) were the dartboard players, and the other two were spectators. Participants were told that they would watch a darts match between the two avatar players. In order to start the game, the participant had to pick up one of the two darts. Each of the darts players received three darts to collect as many points as they could (no doubles or triples were included) with a maximum of 60 points. The total score was then subtracted from the 120 base points visualized on a scoreboard above the dartboard. Both darters threw until one of them won the match by having no points left. In the proactive condition, participants were merely instructed to watch the dart game. In the reactive condition, participants were told that they had to pick which player (i.e., the one with the blue or red shirt) they believed would win the game. They were told that they would win an extra 5 EUR in case their selected player would win the game. Participants were instructed to count how many points each darter threw. The VR task was developed in such a way that the participant’s selected player lost the game because the other player cheated. Specifically, the cheating darter pretended to drop one of the darts that he had to pick off the dartboard. After a small quarrel, the cheater tried to persuade the other darter that he had thrown fewer points and, thus, won the game. Note that the avatar’s height was preprogrammed to have the same height as the individual height of the participant. This reactive version of the VR task bears some similarity to a study of Hubbard et al. [88], where participants lost a board game because of a cheating opponent, and to a VR task developed by Rovira et al. [22], which had participants observing a quarrel about football in a bar, with a verbally provoking avatar. In part 2 of the reactive condition, the participant was informed that he was in the same bar again in order to get another drink, an hour after the darting game had ended. Then, the cheating avatar appeared in front of the participant and walked toward him. The avatar carried the money he won from the game in his shirt pocket (see Figure 5), visible for the participant. Next, the avatar provoked the participant by saying “Didn’t you bet on the other guy?”, “You did, didn’t you! How does it feel to lose?”, “You’re a loser for betting on the other guy!”, “What are you going to do about it, huh?”, “Do you want to punch me?”, “Come on, punch me!”, “You’re too afraid, aren’t you?”, “You’re pathetic”. After a few minutes, the avatar stopped provoking and left the room. During this time, the participant could either do nothing, verbally respond, or hit the avatar. In case the avatar was struck, he fought back (see Figure 5, left). The behavioral outcome of the reactive VR condition was the degree of physical aggression, calculating by counting the number of hits on the avatar’s torso and head. In the proactive condition of part 2, the participant was also informed that he was in the same bar again in order to get another drink, an hour after the darting game had ended. The winning avatar was standing next to the participant. The participant then was provided with three choices (see Figure 6). First, challenge the avatar to a darting game for his money. The participant would receive 50 cents per thrown dart, no matter the outcome, with a maximum of 3 EUR. Second, start a fistfight with the avatar trying to steal the money he won, for which the participant would receive 6 EUR. Third, take the bottle on the table and hit the avatar with it, thereby assuring that the participant could take all of the avatar’s money receiving 10 EUR. The participant was told that some choices made would involve some risk (as it would in the real world). After this explanation, the participant was asked to perform this action of choice. The behavioral outcomes of the proactive VR condition included the choice made by the participant. In the case of choice two, the level of physical aggression was determined by counting the number of hits on the avatar’s torso and head.

Aggression Questionnaire (AQ) [97]. The AQ consists of 29 items assessed through a five-point Likert-type scale (1 = “completely disagree” to 5 = “completely agree”), which correspond to four subscales of aggression; physical aggression (nine items), verbal aggression (five items), anger (seven items), and hostility (eight items). This four-factor structure was confirmed through factor analysis in previous studies, and psychometric studies further found internal consistency values between α = 0.51 and 0.82 and good test–retest reliability [98,99]. In the present sample, only the AQ total was assessed, with α = 0.71 in the current sample. 

For details on the Reactive–Proactive Questionnaire (RPQ), see study 1. In the present sample, α = 0.76 for reactive, α = 0.72 for proactive aggression, and α = 0.83 for total aggression.

Psychopathic traits were assessed using the Dutch version of the Psychopathic Personality Inventory Revised (PPI-R); see study 1 for details. In the present sample, α = 0.92 for PPI-R total, α = 0.91 for PPI FD, α = 0.85 for PPI SCI, and α = 0.77 for PPI Cold-heartedness.

Emotional states were assessed using the modified Differential Emotions Scale (mDES) [100,101]. The mDES measures discrete emotional dimensions, using 16 items scored on a seven-point Likert scale (from 1 = “not at all” to 7 = “very intense”). The mDES consists of two subscales, positive emotions and negative emotions, with nine and seven items, respectively. Psychometric analyses of previous studies showed that both subscales displayed satisfactory internal validity (α = 0.79 for positive emotions; α = 0.69 for negative emotions) [101], and the scales also showed adequate construct and criterion validity, as well as satisfactory reliability [102]. In the present sample, negative emotions and anger were assessed, with α = 0.79 for the pre-measure and α = 0.74 for the post-measure.

Systolic and diastolic blood pressure were measured four times at each assessment using a sphygmomanometer Omron M5-I (Omron Matsusaka Co. Ltd., Matsusaka, Japan) via a standard cuff placed on the subject’s right arm above the elbow.

Five questions were asked to assess how immersed participants were in the VR environment (see study 1; the first five questions were asked).

#### 3.1.3. Procedure

This between-subject study was conducted in accordance with the Declaration of Helsinki and approved by the Ethical Committee of Maastricht University (approval code ECP 23_02_2014). Participants were recruited via advertisements at university billboards. Upon arrival at the VR lab, participants were informed about the general study procedures. After signing informed consent, participants completed autobiographic questions and filled out the baseline mDES measure using an online computer program. Next, the baseline level of the participant’s blood pressure was measured four times and averaged. Participants were then randomly assigned to either first filling out the trait measurements (i.e., PPI-R, AVL, RPQ) or first completing the VR experiment. The main measures were counterbalanced in sequence (condition A: PPI-R, AQ, RPQ; condition B: AQ, RPQ, PPI-R, condition C: RPQ, PPI-R, AQ). Participants were randomly assigned to either the reactive or the proactive VR aggression condition. The VR lab specifications of this study were similar to those of study 1, with the exception that the participant’s hands were now visualized by giving a Razor Hydra gaming controller to the participant which consisted of two motion sensing controllers, allowing both hands to be used individually. After completing the VR experiment, blood pressure was measured again, and participants filled out the post-mDES measurement, as well as the VR questionnaire. Finally, an exit interview was administered to verify whether the participants were aware of the true purpose of the study. Directly after the end of the experiment, the written debriefing was handed out and read aloud by the experimenter. On request of the ethical committee, it was explicitly noted that aggressive behavior is not tolerable or desirable in everyday situations. The participant was thanked for participating. Psychology undergraduates received credit points in return for their participation. All other participants received a monetary reward voucher.

### 3.2. Results 

Table 4 provides the descriptive statistics of all study variables. Mann–Whitney U tests were used to assess group differences because of non-normal data distribution. Results shows that both groups did not significantly differ in any of the trait (i.e., aggression, psychopathy) or baseline (i.e., affect, blood pressure) variables, evidencing the comparability between participants of both conditions. The outcomes of the VR questionnaire indicated that nausea levels were again low, similar to those of study 1. Authenticity of the VR environment and avatars and telepresence were rated between 35 and 71/100 and, thus, comparable to earlier VR studies [78,79,80,83,84,85]. Compared to study 1, avatar authenticity and VR world feelings were rated higher, *t*(71) = 3.75, *p* < 0.001 and *t*(71) = 1.97, *p* = 0.05, respectively. The losing avatar was rated significantly less friendly compared to the winning avatar, *t*(48) = 3.62, *p* = 0.001, and the winner was perceived significantly more friendly in the proactive condition.

*t*-Tests showed that self-reported anger significantly increased from pre- to post-measure in the total sample (Table 5). Anger significantly increased in the reactive aggression condition (Table 5). Analyses showed that systolic blood pressure significantly increased from baseline to post-measure in the total group, but just failed to reach significance within both conditions separately (Table 5). 

Twenty-one participants (80.77%) of those assigned to the reactive aggression VR condition did not aggress in the VR environment. The remaining five participants (19.23%) hit the provoking avatar between seven and 35 times (M = 22.80, SD = 11.05). Because this count variable was strongly negatively skewed, we used negative binomial regression analyses with the number of hits rescaled to decimal categories as the dependent variable, along with self-reported aggression, psychopathy, emotions, and blood pressure as covariates. Table 6 shows that the degree of reactive aggression displayed by those in the reactive VR condition correlated significantly positively with total, verbal, and hostility self-reported aggression scores obtained on the AQ, as well as with PPI-R total and FD factor scores. Nineteen participants (79.2%) of those assigned to the proactive VR condition did not aggress in the VR environment. The remaining five participants (20.9%) hit the provoking avatar between 17 and 60 times (M = 29.20, SD = 17.60), either with the hand (*n* = 1) or with the bottle (*n* = 4). Table 6 shows that the degree of proactive aggression of those in the proactive VR condition was not significantly related to any of the predictors.

### 3.3. Discussion Study 2

Findings show that the bar VR paradigm outperformed the alley VR version (study 1) in four ways. Firstly, participants reported increased authenticity of the VR and higher immersion. This is important because immersion was shown to positively impact the effectiveness of virtual treatments [103]. Secondly, the majority (around 80%) of participants refrained from aggression, while a reverse pattern was seen in the alley VR version. Such lower aggression levels in the VR task evidence the validity of the bar VR task, as the majority of people would also not aggress in real life. Lower aggression levels in study 2 can probably largely be ascribed to the fact that (in contrast to study 1) participants actually had the opportunity to refrain from aggressing while still being active (i.e., throwing darts) in the PA condition. An additional explanation is the between-subject design of study 2, avoiding carryover effects of aggression. Thirdly, there is some evidence for convergent validity of the reactive condition in the VR bar task because of positive correlations between VR displayed aggression and trait levels of aggression (AQ) and psychopathy FD factor and total scores. Similarly, reactive VR aggression showed the expected positive correlations with increased changes in self-reported state anger. Lastly, VR assessed reactive aggression just failed to show the expected increase in systolic blood pressure. 

Self-reported reactive aggression did not correlate with VR assessed reactive aggression. Apparently, while VR reactive aggression does share overlap with self-reported aggression in general, this was not the case at the differential motivational level. On the one hand, such a lack of convergence can be considered unexpected [96]. On the other hand, such discrepancies have previously been found with other behavioral aggression measures, such as the RPQ and the Competitive Reaction Time Task [104,105,106]. One highly plausible explanation for this lack of correspondence is that questionnaires and behavioral measures assess fundamentally different units of analyses. Specifically, questionnaires assess trait aggression, expressed both verbally and physically, often mixed with anger or hostility [16]. In contrast, our VR measure assessed state physical aggression. Furthermore, socially desirable answer tendencies likely influence self-report of aggression [11], causing the latter to deviate from behavioral tasks. 

The psychometric properties of the proactive aggression variant of our VR bar paradigm received less support. VR-assessed and self-report levels of aggression were uncorrelated, and VR-assessed proactive aggression was unrelated to psychopathy levels, contrasting studies evidencing a link between proactive aggressive behavior and the FD and SCI subscales of the PPI-R [107,108].

## 4. General Discussion

Validly assessing aggression is challenging because self-report measures present participants with hypothetical and trait-like tendencies which are often distorted by negative response tendencies, while behavioral paradigms often lack ecological validity. VR-based aggression assessment can be experimentally controlled while mirroring real-life situations, thereby ensuring both internal and ecological validity. The current study, therefore, developed and tested a new VR paradigm for the assessment of reactive and proactive aggression. Here, VR was used for the assessment of physical aggression, which may help overcome problems with earlier behavioral aggression paradigms through elimination of cover stories and ethical constraints. 

The first VR task featured a street alley, with the encounter of a provoking versus a non-provoking aggressor to assess reactive and proactive aggression, respectively. While reactive aggression displayed in this VR task correlated positively with self-reported reactive aggression, partly evidencing construct validity, VR aggression proved to be unrealistically high (i.e., ranging between 75% and 100%) and VR emergence levels were unsatisfactory. Because of these drawbacks, we developed a new VR task, now in a bar environment, where participants in the reactive aggression condition were confronted with an avatar who cheated on a darts game, because of which the participants lost money. In the proactive version, participants merely watched a darts game played fair, but later on were provided with the opportunity to physically aggress toward a player-avatar to increase financial gain.

In general, findings on the bar VR paradigm were promising because participants reported increased authenticity of the VR environment and higher immersion. Furthermore, only a minority (around 20%) of participants aggressed in the reactive and proactive aggression conditions, supporting its ecological validity. The fact that about the same percentage of participants (i.e., 20%) aggressed in the reactive and proactive aggression conditions seems unexpected because, in general, people tend to report that they are more inclined to show reactive than proactive aggression [9,94,96,109]. We mostly expected a lower percentage of proactive aggression. Note that the higher percentage of subjects showing aggression in the reactive alley VR paradigm compared to the bar VR paradigm could also reflect a stronger reaction to physical versus verbal assault. Our VR design, however, does not allow disentangling this, as the avatar in the reactive bar VR task punched back after receiving a first punch from the participant, and it would be advisable for future studies follow up on this. 

There was quite some evidence suggesting good construct validity of the reactive aggression VR bar task. Firstly, it positively related to trait levels of self-reported aggression as measured with the AQ. This suggests that VR-assessed and self-reported reactive aggression tap into shared concepts. Secondly, VR-assessed reactive aggression showed a positive relationship with psychopathy total scores, as well as with FD factor scores, which is in line with previous studies [74]. This implies that especially personality traits such as dominance, emotional stability, and venturesomeness contribute to the use of aggression in an impulsive, defensive way. Thirdly, VR-assessed reactive aggression was shown to be uniquely related to increased levels of anger. Its relationship to increased physiological responses in the form of (systolic) blood pressure just failed to reach significance. Future studies with a larger sample should further investigate this, as such a positive relationship would be in line with theoretical accounts designating reactive aggression as the ‘hot-blooded’ counterpart of proactive aggression [93,95] and with several empirical studies [8,88]. The finding of stronger links between VR-assessed aggression and the physiological indices of systolic blood pressure does not come as a surprise, as studies consistently identify systolic blood pressure as a particularly sensitive measure of emotional change in general [110] and anger in particular [111,112].

Taken together, while results provide preliminary and partial support that the bar VR paradigm validly assessed reactive aggression, the proactive aggression VR paradigm requires more refinement. Future VR studies aiming to develop valid proactive aggression tasks would profit from a critical evaluation of the presence of four crucial motivational components of proactive aggression, i.e., lack of angry arousal, unprovoked, instrumental motivation, and moral disengagement [113]. One newly developed behavioral paradigm for proactive aggression that was based on these four components is the Reward-Interference Task [113]. While our proactive paradigm was largely evaluated on the first two criteria, we chose not to induce and assess moral disengagement (i.e., denying or weakening one’s intention to harm others). Furthermore, instrumental motivation might have been higher in case the incentive for proactive aggression was altered. Here, the use of proactive aggression rewarded the participants with either 3 (fistfight choice) or 7 (hitting with bottle) extra EUR in participation fee, which might not have been sufficiently rewarding for participants. Alternatively, participants might have doubted whether they would actually receive this extra money, as most are aware of standard research participating fees in laboratory university studies. Furthermore, (small) monetary rewards might not sufficiently motivate people to engage in proactive aggression, as increasing one’s power or prestige might be more potent triggers for proactive aggression [25]. Aside from this, the lack of validity of the proactive VR task might be specific to the current noncriminal sample, and replication in criminal or forensic samples is, therefore, warranted.

To the best of our knowledge, this study pioneered differentially assessing reactive aggression and proactive aggression using VR. A realistic environment (i.e., a bar), and trigger for the reactive aggression condition (i.e., cheating on a game combined with verbal challenging) were offered as the context for aggression, ensuring ecological validity. These strengths should be considered in light of several limitations. Firstly, the small samples used only permitted to detect large effects. Therefore, the current study should primarily be seen as providing a basis for larger-scale replications. Secondly, the current VR paradigm can only be used in a laboratory environment that technically facilitates VR soft- and hardware. Thirdly, the proactive aggression condition of the VR design needs further refinement. Fourthly, our sample consisted solely of male students, thereby limiting its generalizability to the overall population, including older people and females. Previous studies evidenced men to report higher levels of proactive aggression [105] and display more reactive aggression [59,105] in behavioral tasks; thus, the VR aggression task could become a valuable new tool in further delineating gender differences in aggression. Furthermore, aggression was solely operationalized as physical aggression, thereby ignoring, e.g., verbal aggression. As physical aggression can be considered more extreme compared to verbal aggression, this might have prevented a more subtle, fine-grained aggression assessment. Future studies should assess the criterion validity of VR aggression tasks, e.g., by comparing its outcome with other behavioral aggression assessment methods such as the CRTT [12] and the Voodoo Doll Task [27]. Relatedly, future studies would profit from adding a nonaggressive but active control condition to rule out whether the observed changes in blood pressure do not simply reflect changes in arousal, and from supplementing outcomes with autonomic responses such as skin conductance and heart rate. Lastly, although our VR design pioneered a visual representation of the participants’ hands in the VR environment, the hands were rather static. New aggression VR designs can make use of recently developed hand-operator controllers to allow more realistic and dynamic visual hand representation, which will positively impact emergence. 

Potential implications for VR aggression tasks are vast. They can be used to assess relevant correlates and predictors from different theoretical models or as behavioral outcome measures following therapeutic interventions focused on lowering aggression. VR aggression assessment is highly flexible, e.g., through the modifiability of avatar characteristics (e.g., length, skin color, gender) and the proximity between victim and aggressor. VR also holds the potential to be used as an ecologically valid trigger and risk-assessment tool for preparing convicted aggressors to re-enter society. Lastly, presenting people with aggressive VR scenarios can be used as a training tool for professional groups who are confronted with aggressive incidents, such as the police, security guards, and public transport staff. Such training environments can be used to recognize the type of displayed aggression (i.e., reactive or proactive) and to practice appropriate communication and intervention styles toward aggressors [55]. 

## 5. Conclusions

Taken together, the current study developed and partly validated a promising VR tool to assess reactive aggressive behavior. VR tools can provide aggression researchers with a realistic and modifiable environment that may greatly facilitate future research in this area.

## Figures and Tables

**Table 1:** Study 1: Description of study variables, *N* = 24.

	M (SD)
RPQ	
Total	13.20 (5.20)
Reactive	9.88 (3.52)
Proactive	3.33 (2.39)
PPI	
Total	317.13 (27.04)
FD factor	111.04 (9.98)
SCI factor	161.46 (15.62)
Cold-heartedness factor	44.63 (6.42)
VR questionnaire	
Nauseous	11.88 (22.00)
Authenticity VR world	24.58 (21.65)
Authenticity avatar	17.29 (18.98)
Lab vs. VR world feeling	47.54 (30.29)
Telepresence	19.63 (22.29)
Threatening avatar perception	35.58 (26.79)
Friendliness of avatar	18.00 (18.91)

Note: RPQ = Reactive Proactive Questionnaire; PPI = Psychopathic Personality Inventory; FD = fearless dominance; SCI = self-centered impulsivity.

**Table 2:** Study 1: Negative binomial regression analysis outcomes on the relationship between study variables and continuous degree of reactive/proactive aggression displayed in VR.

	Reactive Aggression *N* = 24, Wald Chi-sq (*p*)	Proactive Aggression *N* = 24, Wald Chi-sq (*p*)
RPQ		
Total	5.61 * (0.02)	0.001 (0.98)
Reactive	6.70 * (0.01)	0.00 (0.98)
Proactive	2.43 (0.12)	0.001 (0.98)
PPI-R		
Total	6.69 * (0.01)	0.13 (0.72)
FD factor	3.76 (0.05)	0.39 (0.53)
SCI factor	4.45 (0.04)	0.03 (0.87)
Cold-heartedness factor	7.14 * (0.008)	0.02 (0.89)

Note: FD = fearless dominance; SCI = self-centered impulsivity; * significant at the Benjamini and Yekutieli’s false discovery rate (FDR, corrected *p* level of 0.02 (seven analyses within both aggression outcomes) [86].

**Table 3:** Study 2: Biographical information of the total sample and split by condition.

	Total Sample (*N* = 50)	Reactive Condition (*N* = 26)	Proactive Condition (*N* = 24)	Difference (PA vs. RA) *t*/*X*^2^ (*p*, df = 48)
Age, M (SD)	22.54 (2.84)	22.38 (3.27)	22.71 (2.35)	−0.40 (0.69) ^a^
Nationality				4.59 (0.20) ^b^
Dutch %	64	76.9	50	
German %	4	3.8	4.2	
Belgian %	30	19.2	41.7	
Luxemburg %	2	0	4.2	
Education				0.92 (0.82) ^b^
Middle school %	60	61.5	58.3	
High school %	6	3.8	8.3	
Graduate school%	10	7.7	12.5	
University %	24	26.9	20.8	
Marital Status				2.68 (0.26) ^b^
Married/living together %	4	7.7	0	
Single %	50	42.3	58.3	
In a relationship %	46	50	41.7	
Working Situation				1.95 (0.38) ^b^
Working %	8	11.5	4.2	
Student %	90	84.6	95.8	
Searching for job %	2	3.8	0	

Note: ^a^ *t*-test, df = 48, ^b^ *X*^2^-test, df = 3.

**Table 4:** Study 2: Description of study variables.

	Total Sample *N* = 50, M (SD)	Reactive Condition *N* = 26, M (SD)	Proactive Condition *N* = 24, M (SD)	Difference (PA vs. RA) Df = 48, MW-U (*p*)
AQ total	77.68 (10.29)	77.73 (8.54)	77.62 (12.09)	306.50 (0.92)
RPQ				
Total	12.30 (5.54)	12.15 (5.50)	12.46 (5.71)	313.00 (0.98)
Reactive	9.18 (3.57)	9.00 (3.57)	9.37 (6.63)	322.00 (0.85)
Proactive	3.12 (2.67)	3.15 (2.72)	3.08 (2.67)	306.50 (0.91)
PPI-R				
Total	318.08 (20.57)	320.62 (22.06)	315.33 (18.91)	267.50 (0.39)
FD factor	108.94 (7.32)	109.54 (7.81)	108.29 (6.84)	282.50 (0.57)
SCI factor	165.40 (14.26)	167.08 (15.76)	163.58 (12.51)	266.00 (0.37)
Cold-heartedness factor	43.74 (5.00)	44.00 (4.62)	43.46 (5.46)	303.50 (0.87)
VR questionnaire				
Nauseous	9.88 (16.31) ^a^	11.40 (16.03) ^b^	8.29 (16.80)	257.50 (0.38)
Authenticity VR world	35.22 (24.57) ^a^	35.96 (21.46) ^b^	34.46 (27.89)	275.00 (0.62)
Authenticity avatar	38.37 (24.06) ^a^	38.84 (31.88) ^b^	36.83 (26.52)	275.50 (0.62)
‘Real’ feeling	44.39 (27.39) ^a^	46.72 (26.63) ^b^	41.95 (28.52)	268.50 (0.53)
Lab vs. VR world feeling	61.27 (26.71) ^a^	61.40 (23.98) ^b^	61.12 (29.81)	317.00 (0.73)
Facility to immerge	71.96 (21.70) ^a^	67.08 (22.90) ^b^	77.04 (19.57)	380.50 (0.11)
Friendliness winner	30.53 (47.95) ^a^	4.84 (51.60) ^b^	57.29 (23.54)	520.00 * (<0.001)
Friendliness loser	62.63 (25.32) ^a^	67.56 (30.09) ^b^	57.50 (18.42)	222.00 (0.11)

Note: ^a^ *N* = 49. ^b^ *N*= 25; AQ = Aggression Questionnaire; RPQ = Reactive Proactive Questionnaire; PPI-R = Psychopathic Personality Inventory Revised; FD = fearless dominance; SCI = self-centered impulsivity; MW-U = Mann–Whitney U tests; * significant at the Benjamini and Yekutieli’s false discovery rate (FDR) corrected *p* level of 0.01 (16 comparison analyses within both aggression outcomes) [86].

**Table 5:** Study 2: Changes, mean, and SD in pre–post affect and blood pressure.

		Total Sample			Reactive Condition			Proactive Condition	
	Pre	Post	*t*-Test (*p*)	Pre	Post	*t*-Test (*p*)	Pre	Post	*t*-Test (*p*)
Negative affect	12.66 (4.60)	13.46 (5.36)	0.95 (0.35) ^a^	12.08 (3.60)	15.08 (6.11)	2.30 (0.03) ^c^	13.29 (5.49)	11.71 (3.79)	−1.90 (0.07) ^e^
Anger	1.34 (0.80)	1.98 (1.50)	2.66 * (0.01)	1.19 (0.40)	2.46 (1.81)	3.55* (0.002)	1.50 (1.06)	1.46 (0.84)	−0.16 (0.87)
Systolic BP	124.13 (12.99)	127.21 (13.08)	3.04 * (0.004) ^b^	127.08 (13.50)	129.89 (13.72)	2.24 (0.04) ^e^	121.18 (12.01)	124.53 (12.11)	2.07 (0.05) ^e^
Diastolic BP	76.36 (8.18)	76.98 (9.65)	0.79 (0.43) ^b^	75.98 (7.77)	76.89 (9.74)	0.98 (0.34) ^e^	76.74 (8.72)	77.08 (9.76)	0.27 (0.79) ^e^

Note: ^a^ df = 49. ^b^ df = 47. ^c^ df = 25. ^d^ df = 24. ^e^ df = 23; some participants’ blood pressure data were missing due to technical difficulties; BP = blood pressure; * significant at the Benjamini and Yekutieli’s false discovery rate (FDR corrected *p* level of 0.02 (four comparison analyses within both aggression outcomes) [86].

**Table 6:** Study 2: Negative binomial regression analyses outcomes on the relationship between study variables and continuous degree of reactive/proactive aggression displayed in VR.

	Reactive Aggression *N* = 26 Wald Chi-sq (*p*)	Proactive Aggression *N* = 24 Wald Chi-sq (*p*)
AQ	5.55 * (0.018)	1.07 (0.30)
RPQ		
Total	0.98 (0.32)	0.65 (0.42)
Reactive	1.80 (0.18)	0.41 (0.52)
Proactive	0.08 (0.77)	0.63 (0.43)
PPI-R		
Total	5.67 * (0.017)	0.24 (0.63)
FD factor	6.90 * (0.009)	0.01 (0.93)
SCI factor	0.28 (0.10)	1.72 (0.19)
Cold-heartedness factor	0.11 (0.75)	1.69 (0.19)
mDES difference		
Negative	0.29 (0.59)	4.81 (0.03)
Anger	0.90 (0.34)	0.003 (0.95)
Blood pressure difference		
Systolic	1.68 (0.20)	4.22 (0.04)
Diastolic	2.10 (0.14)	1.10 (0.30)

Note: FD = fearless dominance; SCI = self-centered impulsivity; * significant at the Benjamini and Yekutieli’s false discovery rate (FDR) corrected *p* level of 0.02 (12 analyses within both aggression outcomes) [86].

## Data Availability

Data supporting reported results can be found in OSF at 10.17605/OSF.IO/D8YBE [doi], registration number https://osf.io/nm2s8 (accessed on 14 October 2021 ).
